# Moderate ischemic mitral regurgitation (IMR) and metabolic syndrome: where are we now and where are we going?

**DOI:** 10.1186/1475-2840-5-24

**Published:** 2006-11-13

**Authors:** Khalid A Osman, Mohamed H Ahmed

**Affiliations:** 1 Department of Surgery, Queen's Hospital, Burton on Trent, Staffordshire, UK; 2 Chemical Pathology Department, Southampton General Hospital, Southampton, UK

## Abstract

**Background:**

The metabolic syndrome appears to affect 10% to 25% of adult population worldwide. Several studies have described the association between metabolic syndrome and ischaemic heart disease, however, none linked metabolic syndrome to ischemic mitral regurgitation, a serious clinical problem facing both the cardiologists and cardiac surgeons. Ischemic mitral regurgitation is mitral insufficiency caused by myocardial infarction. The myocardial ischemia can result in altered ventricular geometry, leading to mitral insufficiency. Interestingly metabolic syndrome showed more pronounced alteration of left ventricular geometry and function especially in obese subjects.

**Presentation of the hypothesis:**

We have recently proposed that there is link between metabolic syndrome and ischemic mitral regurgitation and associated complications. Operative strategy for moderate ischaemic mitral regurgitation continues to be debated between revascularisation alone and concomitant valve repair at the time of coronary artery bypass surgery. Each of the above group has published studies, with results supporting each argument.

**Testing the hypothesis:**

Generally speaking the treatments available for metabolic syndrome are based in both life style modification (dietary advice and advice to increase physical activity) and medical treatment to enhance insulin sensitivity. Randomised controlled trials may show whether the current available treatment of metabolic syndrome may have an impact on moderate ischemic mitral regurgitation.

**Implications of the hypothesis:**

Metabolic syndrome was shown to alter left ventricular geometry and therefore it is possible to postulate that the variation in the response of different patients with moderate ischemic mitral regurgitation to current management may be attributed to the absence and presence of metabolic syndrome. Research testing of this hypothesis in the future may reveal whether concomitant treatment of metabolic syndrome will play part in the management of moderate ischemic mitral regurgitation.

## Background

The clustering of insulin resistance, dysglycemia, dyslipidemia, hypertension and central obesity represent the major features of metabolic syndrome. These clusters of factors may share common etiology and each of which is a risk factor for cardiovascular disease. The metabolic syndrome appears to affect 10% to 25% of adult populations worldwide. Several studies have described the association between metabolic syndrome, diabetes and cardiovascular disease. Definitions of the metabolic syndrome that also include a measure of central obesity have been developed between 1999 and 2001 by the World Health Organization (WHO Consultation, 1999), The European Group for the Study of Insulin Resistance and the National Cholesterol Education Program (NCEP). Recently the International Diabetes Federation produced worldwide consensus definition of the metabolic syndrome in 2005. The criteria for this definition are a waist circumference of ≥ 94 cm for European men and ≥ 80 cm for European women (With lower cut-points for some other ethnic groups) and two or more of the following: Blood pressure, triglyceride and HDL-cholesterol cut-points as for the ATP-III definitions and fasting plasma glucose 5.6 mmol/L [1,2]_._

## Presentation of the hypothesis

A search of the Medline to date has shown that several studies addressed the link between metabolic syndrome and ischemic heart disease (IHD). However, none linked metabolic syndrome to ischemic mitral regurgitation (IMR), a serious clinical problem facing both the cardiologists and cardiac surgeons. Ischemic mitral regurgitation is mitral insufficiency caused by myocardial infarction. Myocardial infarction always precedes IMR. The leaflets and subvalvular apparatus are by definition normal. The disease must be distinguished from mitral regurgitation (MR) associated with coronary artery disease in which no cause and effect relationship exists. Mitral regurgitation associated with dilated cardiomyopathy and profound left ventricular (LV) dysfunction is a related phenomenon but should be considered etiologically distinct from IMR. Ischemic mitral regurgitation is a serious and important complication that adversely affects the patient's prognosis in the chronic post-myocardial infarction phase [3]. The myocardial ischemia can result in altered ventricular geometry causing the inferioposterior wall to bulge outward, displacing the attached papillary muscles apically and outward. The leaflets, tethered at both end, cannot close effectively and are restrained within the left ventricle; this effect is compounded by a decrease in the ventricular force to close the leaflet thus causing IMR. It is associated with higher cardiovascular mortality within 5 years if left uncorrected [3].

Patients with metabolic syndrome represent a high-risk group for coronary heart disease as well as for type II diabetes. Interestingly metabolic syndrome showed more pronounced alteration of left ventricular geometry and function (increased relative wall thickness of LV dimension and mass, left atrial diameter and decreased mitral E/A ratio). From multiple regressions analysis metabolic syndrome was the main independent determinant of LV mass index [4-6]. Further analysis of the Strong Heart Study showed that in over-weight and obese adolescents there was an increase in LV mass, while obesity was associated with mildly reduced LV myocardial performance and increased left atrial force to contribute to LV filling [6]. We have recently proposed that there is link between metabolic syndrome and IMR and associated complications [2].

Importantly, Hickey et al and the SAVE (Survival and Ventricular Enlargement) Study, showed the prevalence of IMR was around 19 % [7,8]. It is well established that mitral valve repair or replacement is of significant benefit in patients with severe IMR, while those with mild IMR are generally offered coronary artery bypass grafting (CABG) alone, with acceptable early and late results [4]. However, in cases of moderate ischaemic mitral regurgitation operative strategy continues to be debated between revascularisation alone and concomitant valve repair.

There are two schools of thoughts regarding the surgical strategies for the treatment of moderate IMR. The first school, advocate more liberal use of annuloplasty techniques to repair the mitral valve at the time of revascularisation. The rationale for such treatment; firstly it was reported that CABG alone for moderate IMR leaves many patients with significant residual MR [9]. Secondly, Mallidi et al 2004, has shown that patients with uncorrected mild to moderate IMR at the time of CABG had a poorer event-free survival, worse late functional status and worsening in the degree of mitral regurgitation [10]. Thirdly, Bolling et al has reported that nearly all patients undergoing mitral valve repair at the time of CABG had improvement in their angina status [11]. Finally in a retrospective study of 467 patients with moderate IMR who underwent CABG alone, moderate ischemic mitral regurgitation did not reliably resolve with CABG surgery alone and it was associated with significantly reduced survival [12].

On the other hand, surgeons who favour CABG alone to treat moderate IMR present the following arguments. First: the Emory University research group and other investigators concluded that excellent hospital survival and long-term functional stability was achieved with CABG alone. Secondly, it was reported that patients who undergo ring annuloplasty for IMR often have persistent or late recurrence of their MR associated with continued LV remodeling and this may be difficult to treat [13]. Thirdly, addition of mitral valve procedure at the time of CABG significantly increases operative mortality and reduces long-term survival [14]. Finally, redo surgery carries significant risks.

None of the above studies mentioned whether the presence of metabolic syndrome may have determinant impact on patients who are offered CABG or subjected to surgical correction of moderate IMR. Metabolic syndrome was shown to alter LV geometry and therefore it is possible to postulate that the variation in the response of different patients with moderate IMR to current management may be attributed to the absence and presence of metabolic syndrome.

## Testing the hypothesis

Generally speaking the treatments available for metabolic syndrome are based in both life style modification (dietary advice and advice to increase physical activity) and medical treatment to enhance insulin sensitivity. Randomised clinical trial may show whether the current available treatment of metabolic syndrome may have an impact on moderate ischemic mitral regurgitation.

## Implications of the hypothesis

Research testing of this hypotheses in the future may reveal whether concomitant treatment of metabolic syndrome will also play part in the management of moderate IMR.

## Abbreviations

ischemic heart disease (IHD)

ischemic mitral regurgitation (IMR)

Left Ventricle (LV)

coronary artery bypass grafting (CABG)

mitral regurgitation (MR)

## Competing interests

The author(s) declare that they have no competing interests.

## Authors' contributions

The authors have equally made substantial contributions to conception and design of the hypothesis, drafting the manuscript, revising it critically for important intellectual content; and have given final approval of the version to be published.
